# Identification of common diagnostic biomarkers and immune landscapes in sepsis and acute kidney injury: a transcriptomic study integrating machine learning and single-cell analysis

**DOI:** 10.3389/fgene.2026.1837345

**Published:** 2026-07-22

**Authors:** Yeqiu Huang, Shengnan Fei, Minmin Huang, Xinzhong Huang, Fei Lu

**Affiliations:** 1 Department of Nephrology, Affiliated Hospital of Nantong University and Medical School of Nantong University, Nantong, China; 2 Department of Critical Care Medicine, Haimen District People’s Hospital, Nantong, China

**Keywords:** acute kidney injury, immune microenvironment, machine learning, sepsis, single-cell RNA sequencing

## Abstract

**Background:**

Sepsis and acute kidney injury (AKI) are life-threatening conditions often coexisting as sepsis-associated AKI (S-AKI). However, their shared molecular mechanisms and immune heterogeneity remain unclear. This study aims to identify robust diagnostic biomarkers applicable to both conditions and to elucidate their diverse immune microenvironments using integrated transcriptomic approaches.

**Methods:**

Transcriptomic datasets for sepsis and AKI were analyzed, with multiple cohorts used for training and external validation. Differential gene expression and WGCNA identified key modules, while LASSO and Random Forest algorithms screened shared hub genes. A diagnostic nomogram was constructed and evaluated using ROC and decision curve analyses. Single-cell RNA sequencing data were further analyzed to determine cellular localization, functional pathways, and intercellular communication.

**Results:**

Four hub genes (*FBXO21*, *FLOT1*, *TMC6*, and *KLRB1*) were identified as robust diagnostic biomarkers for both sepsis and AKI, demonstrating strong predictive performance across validation cohorts. Single-cell analysis revealed that these genes were enriched in specific immune cell populations and injured renal cells, and were closely associated with T-cell activation and immune signaling pathways. Cell-cell communication analysis further inferred distinct ligand–receptor interactions that may underlie immune crosstalk in both conditions.

**Conclusion:**

We identified a reliable four-gene diagnostic signature shared by sepsis and AKI and characterized their shared immune heterogeneity and inferred intercellular communication networks. These findings provide potential targets for early diagnosis and therapeutic intervention in sepsis-associated renal injury.

## 1 Introduction

Acute Kidney Injury (AKI) and sepsis represent two of the most formidable challenges in contemporary critical care, collectively contributing to a staggering global health burden. AKI, characterized by a sudden impairment of renal excretory function and acid-base homeostasis, affects millions of patients annually, with its incidence continuing to rise due to an aging population and increasing clinical complexity (Hoste et al., 2015). Despite the implementation of standardized diagnostic criteria such as the KDIGO guidelines, clinical management remains hampered by the limitations of traditional functional markers. Serum creatinine and urine output, the current “gold standards,” are reactive rather than proactive indicators, often remaining unchanged until more than 50% of kidney function is lost (Peerapornratana et al., 2019). Sepsis, a dysregulated host response to infection leading to life-threatening organ dysfunction, remains a leading cause of death in intensive care units (ICUs) worldwide (Gómez et al., 2017). The intersection of these two conditions creates a synergistic effect that drastically worsens patient prognosis, necessitating a deeper exploration of their shared molecular foundations to move beyond late-stage functional detection toward early, mechanism-based diagnosis. Despite advances in supportive care, the pathophysiological mechanisms of SA-AKI remain incompletely understood, and the lack of early, specific diagnostic biomarkers contributes to poor clinical outcomes (Ostermann et al., 2020). Although AKI and sepsis are distinct pathological entities, they share profound commonalities at the molecular level, particularly regarding the dysregulation of the host immune response (Rudd et al., 2020). Therefore, exploring the shared transcriptomic landscape of AKI and sepsis is crucial for identifying robust common biomarkers and elucidating the convergent molecular mechanisms driving these two interconnected conditions. Although the coexistence and overlapping pathophysiology of sepsis and AKI are now well recognized, S-AKI occurs in nearly half of septic patients and in roughly one in six ICU admissions, and carries higher mortality than either condition alone (Takeuchi et al., 2025; Zarbock et al., 2023), this knowledge has so far remained largely descriptive and has not been translated into shared, host-response-based molecular markers. Clinically, a common signature could be valuable in several respects: it would provide a unified, mechanism-based readout in the ICU setting where the two conditions frequently co-occur; it would not depend on functional indicators such as serum creatinine, which lack specificity and rise only after substantial injury has occurred (Nourie et al., 2024); and, by capturing biology shared across both diseases, it could point toward converging rather than disease-specific therapeutic targets.

The immune system plays a pivotal role in the progression of both AKI and sepsis. In the acute phase, the massive release of damage-associated molecular patterns (DAMPs) and pathogen-associated molecular patterns (PAMPs) triggers a systemic inflammatory storm, leading to collateral tissue damage (Venet and Monneret, 2018). T lymphocytes, particularly the CD8^+^ cytotoxic subset, are the core orchestrators of this response. Recent studies have highlighted that T cell exhaustion and apoptosis are hallmarks of sepsis-induced immunoparalysis, while in the kidney, infiltrating T cells can exacerbate tubular injury via direct cytotoxicity or cytokine release (Hotchkiss et al., 2013; Sato and Yanagita, 2018). Crucially, the functional state of these T cells is increasingly recognized as being governed by their cellular metabolism; however, the specific phenotypic alterations of T cells during the transition from systemic infection to local organ injury remain elusive. Moreover, how metabolic signals within the microenvironment, such as hypoxia-induced metabolites or systemic inflammatory mediators, shape T cell fate differs between the circulation in sepsis and the tissue microenvironment in AKI (Yang et al., 2023). Currently, there is a paucity of studies integrating bulk and single-cell transcriptomics to systematically dissect the crosstalk between metabolic reprogramming and immune signaling under these two conditions (Lech et al., 2014).

Whereas previous molecular studies have largely examined S-AKI as a single entity, typically relying on bulk transcriptomics with deconvolution-based immune estimation (Ye et al., 2025), few have systematically derived and externally validated a cross-disease diagnostic signature applicable to sepsis and AKI separately, or resolved such shared genes at single-cell resolution (Lin et al., 2025; Zhai et al., 2025; Li et al., 2025; Nian et al., 2025). To address this gap, we employed a comprehensive bioinformatics approach to unveil the shared molecular signatures and immune heterogeneity underlying AKI and sepsis. By integrating Weighted Gene Co-expression Network Analysis (WGCNA) and machine learning algorithms across multiple independent training and external validation cohorts, we identified a robust four-gene diagnostic signature (*FLOT1*, *FBXO21*, *KLRB1*, *TMC6*). Furthermore, utilizing single-cell RNA sequencing (scRNA-seq) analysis, we mapped the cellular localization of these hub genes and revealed their specific enrichment in CD8^+^ T cells. The originality of this work therefore lies in three aspects: (i) it provides a shared, externally validated four-gene diagnostic signature for both sepsis and AKI rather than for a single condition; (ii) it integrates bulk and single-cell transcriptomics to localize this signature to specific T-cell populations; and (iii) it delineates the divergent immune trajectories underlying the shared signature, an *MIF*-axis-associated metabolic-stress phenotype in AKI versus a Resistin-signal-associated cytotoxic/exhausted phenotype in sepsis. This study aims to provide novel insights for diagnostic stratification and identify potential immunometabolic therapeutic targets for patients suffering from AKI and sepsis.

## 2 Methods

### 2.1 Data acquisition

#### 2.1.1 Bulk microarray datasets

For sepsis, two independent microarray datasets were obtained from Gene Expression Omnibus (GEO) database (https://www.ncbi.nlm.nih.gov/geo/) (Table 1). The dataset GSE57065, containing whole blood samples from 25 healthy controls and 82 sepsis patients, was used as the training cohort. GSE13904 (18 controls and 52 sepsis samples), GSE26378 (21 controls and 82 sepsis samples), and GSE95233 (22 controls and 102 sepsis samples) were used as external validation datasets. For acute kidney injury (AKI), multiple GEO datasets were collected. The datasets GSE139061 (9 normal controls and 39 AKI samples) and GSE30718 (19 normal controls and 28 AKI samples) were merged and used as the training cohort. Prior to downstream analysis, batch effects between these two datasets were corrected using the Surrogate Variable Analysis (SVA) algorithm. Three independent datasets were used as external validation cohorts for AKI, including GSE61739 (48 normal controls and 48 AKI samples), GSE67401 (53 normal controls and 58 AKI samples), and GSE217427 (22 normal controls and 22 AKI samples).

**TABLE 1:** Metadata summary of the datasets used in this study.

Dataset ID	Disease	Usage	Platform	Tissue type	Sample size (Control/Disease)
GSE61739	AKI	Bulk	[GPL13158] Affymetrix HT HG-U133+ PM	Kidney	48/48
GSE67401	AKI	Bulk	[GPL9115] Illumina Genome Analyzer II	Whole blood	53/58
GSE217427	AKI	Bulk	[GPL24676] Illumina NovaSeq 6000	Kidney	22/22
GSE30718	AKI	Bulk	[GPL570] Affymetrix U133 Plus 2.0	Kidney	19/28
GSE139061	AKI	Bulk	[GPL20301] Illumina HiSeq 4000	Kidney	9/39
GSE57065	Sepsis	Training (bulk)	[GPL570] Affymetrix U133 Plus 2.0	Whole blood	25/82
GSE13904	Sepsis	Bulk	[GPL570] Affymetrix U133 Plus 2.0	Whole blood	18/52
GSE183276	AKI	scRNA-seq	[GPL24676] Illumina NovaSeq 6000	Kidney tissue	20/12 (AKI)/17 (CKD)
GSE167363	Sepsis	scRNA-seq	[GPL24676] Illumina NovaSeq 6000	PBMC	2/10
GSE175453	Sepsis	scRNA-seq	[GPL24676] Illumina NovaSeq 6000	PBMC	2/7

#### 2.1.2 Single-cell RNA sequencing (scRNA-seq) datasets

For AKI, scRNA-seq data were collected from the GSE183276 dataset, comprising 20 controls, 12 AKI patients, and 17 chronic kidney disease (CKD) patients. For sepsis, scRNA-seq data were collected from the GSE167363 dataset (2 control samples and 10 sepsis samples) and the GSE175453 dataset (2 control samples and 7 sepsis samples), respectively.

### 2.2 Differential gene expression and enrichment analysis

Differential gene expression analysis was performed using the limma (v3.58.1) package in R. Gene expression data were assessed for the need for log transformation based on their distribution, and quantile normalization was applied using the normalize Between Arrays function to reduce technical variability across samples. Specifically, for the AKI dataset, AKI status was modeled as the fixed effect, and the contrast was defined as AKI versus control. For the sepsis dataset, sepsis status was modeled as the fixed effect, and the contrast was defined as sepsis versus control. Probe identifiers were mapped to official gene symbols using the corresponding platform annotation, and probes without valid gene symbols were excluded. For genes represented by multiple probes, the probe with the highest average expression was retained. A linear model was fitted for each gene according to the experimental design, followed by empirical Bayes moderation. P values were adjusted using the Benjamini–Hochberg false discovery rate method, and genes with an adjusted P value <0.05 and |log_2_ fold change| > 1 were defined as differentially expressed genes. Enrichment analysis of DEGs was performed based on the Metascape database (https://metascape.org/gp/).

### 2.3 WGCNA analysis

To explore gene co-expression patterns and their associations with immune infiltration characteristics, weighted gene co-expression network analysis (WGCNA) was performed using the “WGCNA” package (v1.73) in R. WGCNA was conducted on the bulk transcriptomic data. Based on the differential gene expression analysis, 1,103 AKI-related differentially expressed genes were used for WGCNA in the AKI dataset, while 3,046 sepsis-related differentially expressed genes were used for WGCNA in the sepsis dataset. Before network construction, outlier samples were detected and removed through hierarchical clustering. The optimal soft-thresholding power was determined using the pickSoftThreshold function according to the scale-free topology criterion and mean connectivity. Subsequently, an adjacency matrix was constructed and transformed into a topological overlap matrix (TOM), which was used for hierarchical clustering. Gene modules were identified using the dynamic tree cut algorithm with minModuleSize = 30. Similar modules with a module eigengene dissimilarity less than 0.25 were merged. Finally, module eigengenes were correlated with disease phenotypes and immune infiltration characteristics to identify biologically relevant modules.

### 2.4 Machine learning

To ensure the robustness of the prediction models and minimize overfitting, this study adopted a 10-fold cross-validation strategy. In each iteration, the Least Absolute Shrinkage and Selection Operator (LASSO) regression algorithm was first executed within the training set using the “glmnet” package (v4.1) in R for feature selection. The optimal penalty parameter was determined through internal 10-fold cross-validation, and features with non-zero coefficients at lambda.min were selected as key diagnostic variables. In the Random Forest analysis, genes with an importance score greater than 1 were retained as candidate key diagnostic genes. Subsequently, based on these selected features, multivariate Logistic Regression (LR) and Random Forest (RF, containing 1,000 decision trees using the “randomForest” package in R, v4.7) classifiers were constructed respectively. Finally, by integrating the prediction results from all test sets, Receiver Operating Characteristic (ROC) curves were drawn and the Area Under the Curve (AUC) was calculated using the “pROC” package (v1.19.0.1) in R to comprehensively evaluate the predictive performance of the two models in distinguishing different subtypes.

### 2.5 Nomogram model construction and evaluation

To evaluate the clinical application value of key biomarkers in predicting the risk of both diseases, this study constructed a visual diagnostic nomogram based on multivariate logistic regression analysis using the “rms” package (v6.7) in R software. Internal validation was performed using the Bootstrap method with 1,000 resamplings, and calibration curves were plotted to evaluate the consistency between the nomogram-predicted probabilities and actual observations. Subsequently, Decision Curve Analysis (DCA) was performed using the “rmda” (v1.6) package to quantify the clinical utility of the model by calculating the Net Benefit at different threshold probabilities. In addition, Clinical Impact Curves (CIC) were drawn to visually display the relationship between the population deemed high-risk by the model and the actual number of positive patients under different risk thresholds, thereby further comprehensively evaluating the predictive performance of the model.

### 2.6 Immune infiltration analysis

To comprehensively evaluate the abundance of immune cell infiltration within the tissue microenvironment, this study performed single-sample Gene Set Enrichment Analysis (ssGSEA) using the “GSVA” package (v1.50.5) in R software. Based on a predefined immune marker gene set (immune.gmt), enrichment scores for different immune cell populations in each sample were calculated by mapping gene expression profiles to specific immune signatures. To ensure comparability between samples and cell types, a min-max normalization strategy was adopted to process the raw enrichment scores, mapping the scores to a range of 0–1. These standardized scores were used to represent the relative infiltration levels of immune cells for subsequent analysis.

### 2.7 scRNA-seq data analysis

#### 2.7.1 Quality control and cell type identification

Whereas Sections 2.2–2.6 describe the bulk microarray workflow, Sections 2.7.1–2.7.4 describe the independent scRNA-seq workflow applied to the datasets listed above. scRNA-seq data were processed with the Seurat package (v4.4.0) in R (Hao et al., 2021). The objects from the different datasets were merged into a single object, and the mitochondrial gene proportion was quantified per cell with PercentageFeatureSet (genes matching the “^MT-” pattern). For quality control, only cells with 200–4,000 detected genes (nFeature_RNA), 1,000–20,000 total UMI counts, a mitochondrial gene content ≤20% and a ribosomal gene content ≤20% were retained. The filtered count matrix was normalized using the LogNormalize method (scale factor = 10,000), and the top 1,500 highly variable genes (HVGs) were identified with the variance-stabilizing transformation. The data were scaled across all genes, and principal component analysis was performed on the HVGs. To correct for batch effects across the different sample types, the Harmony algorithm was applied to the PCA embedding, using sample type as the grouping variable (Korsunsky et al., 2019). Based on the elbow plot, the first 20 Harmony-corrected dimensions were retained for all downstream steps. A shared nearest-neighbor (SNN) graph was constructed on these 20 dimensions, and cells were clustered with the Louvain algorithm (Subelj and Bajec, 2011). To determine a stable resolution, a range of resolutions (0.01–3) was evaluated and visualized with clustree (Zappia and Oshlack, 2018), and a resolution of 1 was selected for the final clustering. Clusters were projected into two dimensions using UMAP and t-SNE, both computed on the same 20 Harmony dimensions. Cell types were annotated with a hybrid strategy: automated cluster-level annotation was first performed with SingleR (v2.0.0; method = “cluster”) against a human reference dataset using its main cell-type labels (Aran et al., 2019).

#### 2.7.2 Hub-gene signature scoring

To evaluate the overall expression level of the hub-gene signature across cell types, a per-cell module score was computed with the AddModuleScore function of Seurat on the RNA assay (Tirosh et al., 2016). The signature consisted of the four hub genes (*FBXO21*, *FLOT1*, *TMC6*, *KLRB1*), restricted to those detected in the dataset. For each cell, AddModuleScore calculates the average expression of the signature genes and subtracts the average expression of a randomly selected control gene set (ctrl = 100 control genes drawn from expression-matched bins), thereby controlling for differences in baseline expression and cell-to-cell technical variation. The resulting signature scores were compared across cell subpopulations using violin plots (grouped by cell type) and were projected onto the t-SNE embedding as a continuous color gradient. To identify the cell population with the highest signature activity, the median score of each cell type was calculated and the cell types were ranked in descending order; the top-ranked population was taken as the compartment with the greatest signature abundance.

#### 2.7.3 GSEA analysis

To identify the biological functions and signaling pathways enriched in specific T-cell subsets, pre-ranked gene set enrichment analysis (GSEA) was performed independently for each disease using fgsea package (v1.24.0), using CD4^+^ T cells in AKI and CD8^+^ T cells in sepsis as the cell populations of interest. For each disease, the target T-cell subset was compared against all remaining T cells of the same dataset with the FindMarkers function (Wilcoxon rank-sum test) of the Seurat package. To construct the ranked gene list required by GSEA, every detected gene was retained (without any fold-change or significance pre-filtering) and ordered in descending order by its average log2 fold change, so that the ranking metric was the average log2 fold change between the target subset and the remaining T cells. Human KEGG canonical-pathway gene sets (category “C2”, subcategory “CP:KEGG”) were retrieved from the MSigDB database (https://www.gsea-msigdb.org/gsea/msigdb) using the “msigdbr” R package. Pre-ranked GSEA was then executed on this ranked list with the “fgsea” package (1,000 permutations), restricting analysis to gene sets containing between 15 and 500 genes. Pathways with a Benjamini–Hochberg adjusted P-value <0.05 were regarded as significantly enriched. The significantly enriched pathways were ranked by their normalized enrichment score (NES), and the top 20 were displayed as a bar plot.

#### 2.7.4 Cell-cell communication analysis

To infer cell-cell communication networks and identify key signaling pathways, the CellChat package (v2.2.0) in R was employed (Jin et al., 2021). A CellChat object was created from the normalized expression matrix together with the cell-type annotation, using cell type as the grouping variable. The human ligand–receptor reference CellChatDB.human was used, and the database was restricted to the “Secreted Signaling” category to focus on paracrine and autocrine interactions. After subsetting the signaling genes, over-expressed genes and over-expressed ligand–receptor pairs were identified with *identifyOverExpressedGenes* and *identifyOverExpressedInteractions* function, respectively. Communication probabilities were then computed at the ligand-receptor level with *computeCommunProb* function, based on the law of mass action. To ensure robustness, ligand-receptor pairs inferred from cell groups containing fewer than 3 cells were discarded using *filterCommunication* function. Signaling was then aggregated at the pathway level with *computeCommunProbPathway* function, and the overall network was summarized with aggregateNet. The aggregated network was visualized as circle plots showing the number and the strength of interactions among cell types, and specific source to target ligand-receptor pairs were displayed with bubble plots. Network centrality scores were computed with *netAnalysis_computeCentrality* function to determine the dominant senders and receivers, and the expression of the genes involved in the key pathways (*MIF*, *ANXA1/ANNEXIN* and *RESISTIN*) was shown with *plotGeneExpression* function.

### 2.8 Cell culture and model establishment

Human proximal tubular epithelial cells (HK-2) were obtained from the Cell Bank of the Chinese Academy of Sciences (Catalog No. SCSP-5204). The cells were authenticated by STR profiling and confirmed to be mycoplasma-free. Cells were cultured in high-glucose DMEM supplemented with 10% fetal bovine serum (FBS) and 1% penicillin-streptomycin in a humidified incubator at 37 °C with 5% CO_2_. Upon reaching 80%–90% confluence, the culture medium was discarded. The cells were washed twice with PBS and dissociated using 0.25% trypsin-EDTA for 2–3 min. Digestion was terminated by adding complete medium containing 10% FBS when the cells retracted and became rounded. A single-cell suspension was prepared by gentle pipetting, and cells were subcultured at a ratio of 1:3. For the experimental model, cells in the logarithmic growth phase were seeded into 6-well plates at a density of 2 × 1,052 \times 10^52^ × 105 cells/well with 2 mL of medium and incubated for 24 h to allow complete attachment. To establish the sepsis-associated acute kidney injury (AKI) model, cells were treated with Lipopolysaccharide (LPS, *E. coli* O111:B4) at a final concentration of 1 μg/mL combined with 10 μmol/L cisplatin for 24 h. Six replicate wells were set up for each experimental group.

### 2.9 Real-time quantitative PCR (RT-qPCR)

After treatment, the medium was removed, and cells were washed twice with PBS. Total RNA was extracted by adding 1 mL of TRIzol reagent to each well and lysing for 5 min at room temperature. The lysate was collected, mixed with 200 μL of chloroform, shaken vigorously for 15 s, incubated at room temperature for 3 min, and centrifuged at 12,000 rpm for 15 min at 4 °C. The upper aqueous phase was transferred to a new tube, mixed with an equal volume of isopropanol, incubated at −20 °C for 30 min, and centrifuged at 12,000 rpm for 10 min at 4 °C. The supernatant was discarded, and the RNA pellet was washed with 1 mL of 75% RNase-free ethanol. After centrifugation at 7,500 rpm for 5 min at 4 °C, the pellet was air-dried at room temperature and dissolved in RNase-free water. RNA samples were stored at −80 °C.

RNA purity and concentration were assessed using a micro-spectrophotometer (A260/A280 ratio: 1.8–2.0), and integrity was verified by 1% agarose gel electrophoresis. cDNA was synthesized using a reverse transcription kit according to the manufacturer’s instructions (incubation at 42 °C for 60 min, followed by 85 °C for 5 min to terminate the reaction). The cDNA was stored at −20 °C. RT-qPCR was performed in a 20 μL reaction system containing 10 μL of 2× SYBR Green PCR Master Mix, 0.8 μL each of forward and reverse primers (for *FBXO21, FLOT1, TMC6, and KLRB1*), 2 μL of cDNA template, and 6.4 μL of RNase-free water. The thermal cycling conditions were: initial denaturation at 95 °C for 30 s, followed by 40 cycles of 95 °C for 5 s and 60 °C for 30 s. Melting curve analysis was performed to verify specificity. Relative gene expression levels were calculated using the 2^−ΔΔCT^ method. All experiments were performed in triplicate. Table 2 provides the information of the primers. The original, uncropped images of all Western blot membranes are provided in Supplementary Figures S1A–D.

**TABLE 2:** Primer sequence information of hub genes.

Gene	Upstream primer sequence (5′→3′)	Downstream primer sequence (5′→3′)
*FBXO21*	GCC​AGT​TCT​ACG​ACA​TCC​AGA​A	TTG​CTG​CTG​TAG​TCC​TTG​GTG​T
*FLOT1*	TCA​GAC​CTG​GTC​AAC​ATG​G	AGA​GTG​CAA​ATA​GTC​CTG​GTC
*KLRB1*	CAG​AGA​CTT​TGG​TGG​AGA​GCG	ACC​TTG​GGG​GAG​AGA​TGG​A
*TMC6*	GTG​CTG​CTG​AAG​ATG​CTG​TTG	GAC​CGT​GCT​GCT​TCA​TAC​ATG
*β-actin*	CTG​GAA​CGG​TGA​AGG​TGA​CA	AAG​GGA​CTT​CCT​GTA​ACA​ATG​CA

### 2.10 Western blotting

Cells were washed twice with PBS and lysed in RIPA buffer containing 1% protease inhibitors (200 μL/well) on ice for 30 min with repeated pipetting. The lysates were centrifuged at 12,000 rpm for 15 min at 4 °C, and the supernatants were collected as total protein extracts and stored at −80 °C. Protein concentration was determined using a BCA protein assay kit. Equal amounts of protein (20–50 μg) were mixed with 5× SDS loading buffer (4:1 ratio), denatured at 95 °C for 5 min, and separated by 10% SDS-PAGE (80 V for 30 min, followed by 120 V for 90 min). Proteins were transferred onto methanol-activated PVDF membranes using a wet transfer system (100 V for 90 min at 4 °C). The membranes were blocked with 5% non-fat milk for 1 h at room temperature and incubated with primary antibodies against the target proteins and *GAPDH* overnight at 4 °C with gentle shaking. The following day, membranes were washed three times with TBST (10 min each) and incubated with corresponding secondary antibodies for 1 h at room temperature. After three additional washes with TBST, protein bands were visualized using an Enhanced Chemiluminescence (ECL) kit and imaged with a chemiluminescence imaging system. Band intensity was quantified using ImageJ software, with *GAPDH* serving as the internal control. Independent experiments were repeated three times.

## 3 Results

### 3.1 Identification and enrichment analysis of DEGs in AKI and sepsis

**FIGURE 1:**
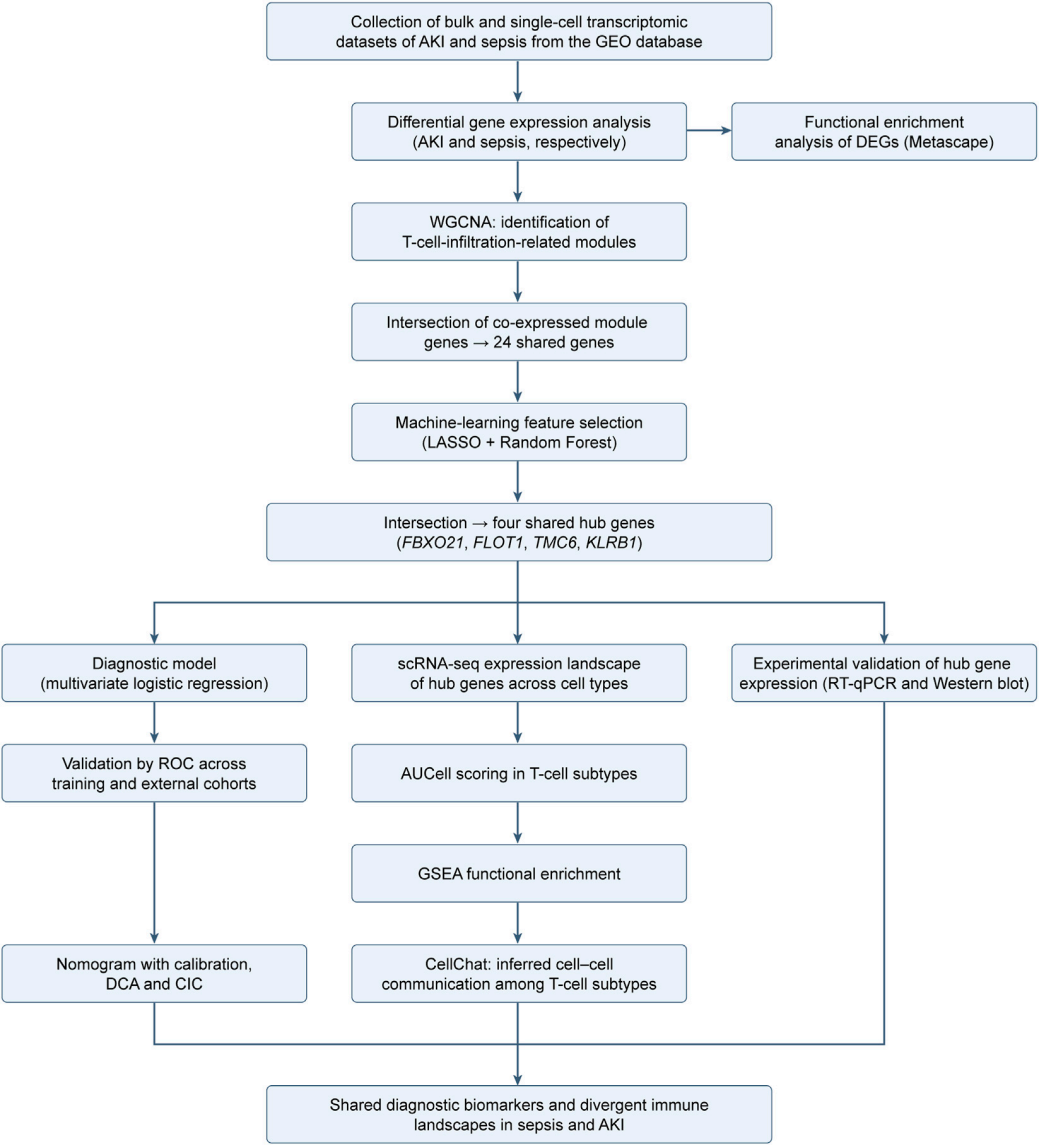
The technical roadmap of this paper.

**FIGURE 2:**
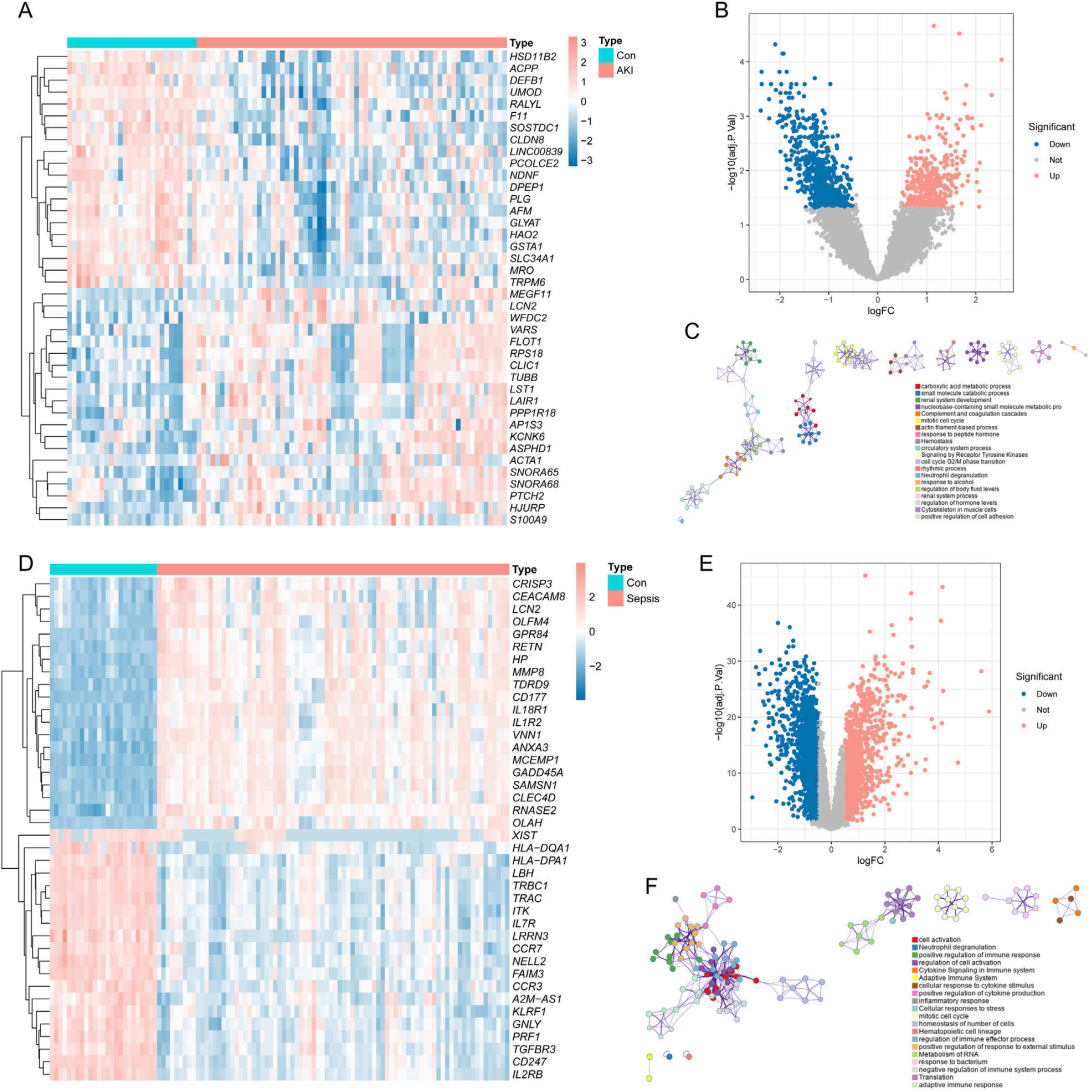
Identification and functional enrichment analysis of DEGs in AKI and sepsis. **(A,D)** Expression heatmaps of the top DEGs in the AKI group **(A)** and sepsis group **(D)** compared with their respective controls. Each column represents a sample, and each row represents a gene. The color intensity reflects the relative level of gene expression (red indicates high expression; blue indicates low expression). **(B,E)** Volcano plots displaying the distribution of DEGs in AKI **(B)** and sepsis **(E)**. The x-axis represents the log2 fold change, and the y-axis represents the negative logarithm (−log10) of the adjusted P-value. Colored points denote significant DEGs (Up: significantly upregulated; Down: significantly downregulated), while gray points represent genes with no significant difference. **(C,F)** Enrichment analysis network diagrams for DEGs in AKI **(C)** and sepsis **(F)** constructed via the Metascape database, illustrating significantly enriched functional pathways and the interaction networks among enriched terms.

To investigate the key molecular alterations and potential biomarkers underlying the pathogenesis of AKI and sepsis, we designed an integrated analytical workflow combining bulk transcriptomics, single-cell transcriptomics, machine learning, and experimental validation (Figure 1). We first conducted differential gene expression analyses in the corresponding AKI and sepsis cohorts. In the AKI cohort, comparison between AKI samples and normal controls identified 1,103 differentially expressed genes based on the criteria of |log_2_FC| > 0.5 and adjusted P < 0.05 (Figures 2A,B; Supplementary Tables S1, S2). Notably, genes involved in maintaining normal renal tubular function, including *UMOD*, *SLC34A1*, *ACPP*, and *DEFB1*, were markedly downregulated in AKI samples. This expression pattern suggests that AKI is accompanied by loss of differentiated tubular epithelial functions, impaired solute transport, and disruption of epithelial homeostasis. The Metascape enrichment network further showed that AKI-related DEGs were mainly enriched in small molecule catabolic process, carboxylic acid metabolic process, and renal system development (Figure 2C). Biologically, these pathways indicate that injured renal tissue undergoes a transition from specialized transport and metabolic functions toward stress-associated metabolic remodeling, which is consistent with tubular injury and maladaptive repair during AKI.

In the sepsis cohort, DEGs showed a distinct immune-dominant pattern (Figures 2D,E). Genes associated with neutrophil activation and innate immune responses, such as *LCN2*, *MMP8*, *RETN*, *CEACAM8*, and *OLFM4*, were prominently upregulated, whereas genes related to adaptive immune function and T-cell receptor signaling, including *IL7R*, *TRAC*, *TRBC1*, *CCR7*, and *ITK*, were downregulated. This pattern reflects a characteristic immunological imbalance in sepsis, namely, simultaneous innate immune hyperactivation and adaptive immune suppression. Enrichment analysis confirmed that sepsis DEGs were mainly involved in neutrophil degranulation, cytokine signaling, and adaptive immune system pathways (Figure 2F). These findings support the concept that AKI and sepsis share immune dysregulation, but AKI is more closely linked to tissue metabolic dysfunction and epithelial injury, whereas sepsis is dominated by systemic innate immune activation and T-cell dysfunction.

### 3.2 Identification of T-cell subtype-related modules and shared genes

**FIGURE 3:**
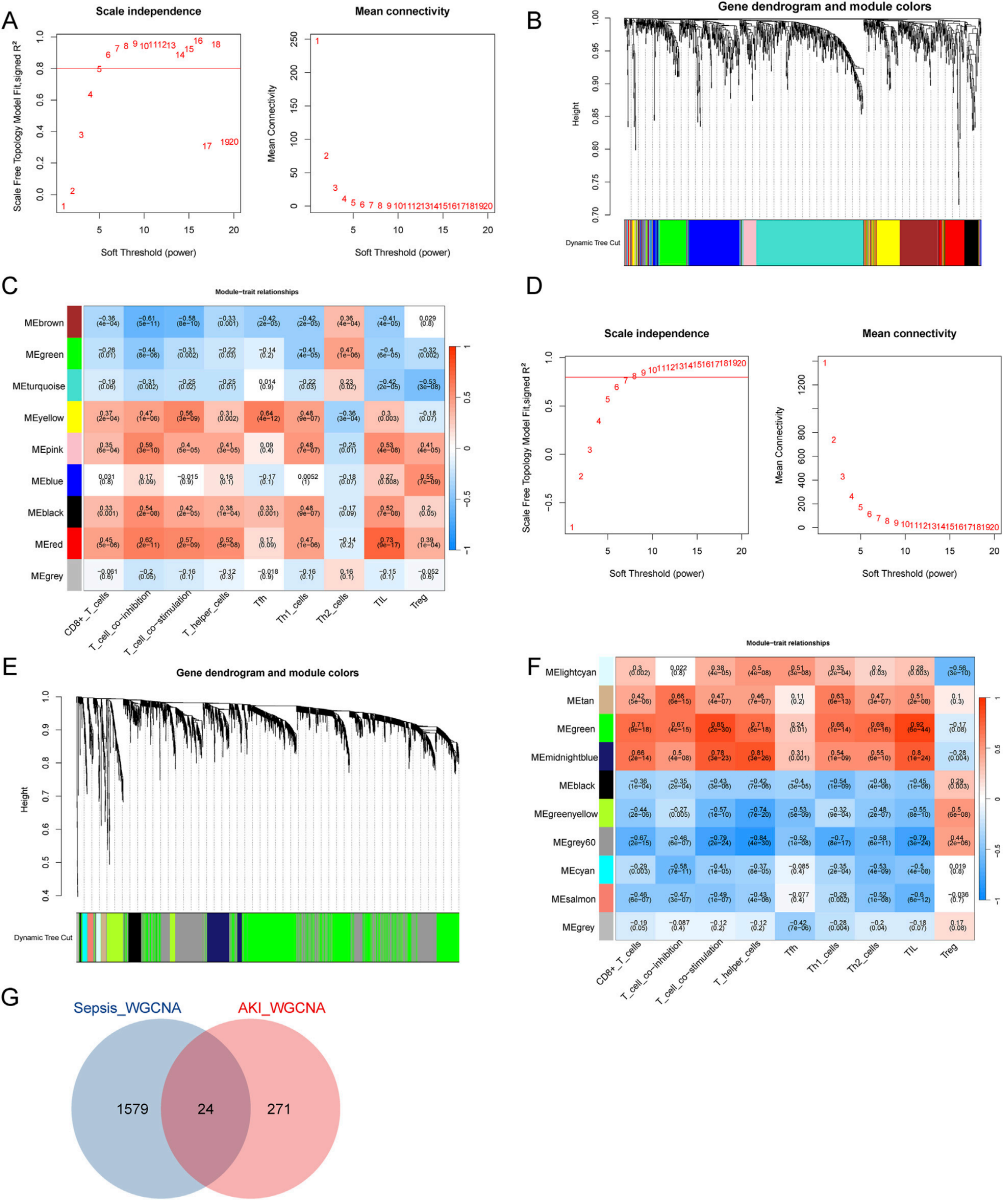
Identification of co-expression modules and shared genes associated with T-cell subtype infiltration abundance/function in AKI and sepsis via WGCNA. **(A,D)** Screening of the soft-thresholding power in the AKI **(A)** and sepsis **(D)** datasets. The left panel plots the scale-free topology fit index against soft-thresholding powers; the red line indicates the cutoff for a scale-free network topology. The right panel displays the relationship between mean connectivity and soft-thresholding powers. **(B,E)** Hierarchical clustering dendrograms of genes constructed based on the Topological Overlap Matrix (TOM) for AKI **(B)** and sepsis **(E)**. The colored rows below the dendrograms represent distinct co-expression modules identified via the dynamic tree cut algorithm. **(C,F)** Heatmaps illustrating the correlation between module eigengenes and T-cell subtype infiltration abundance/functions in AKI **(C)** and sepsis **(F)**. Rows represent module eigengenes, and columns represent distinct T-cell subtypes. The numbers within the cells indicate correlation coefficients, with corresponding P-values in parentheses. Red indicates positive correlation, and green indicates negative correlation. **(G)** Venn diagram displaying the intersection of genes within key modules significantly associated with T-cell infiltration/subtypes in both AKI and sepsis, identifying shared T-cell regulatory genes across the two pathological states.

To further explore the gene co-expression networks closely associated with T-cell immune infiltration, we performed WGCNA on the AKI and sepsis datasets, respectively. Initially, to construct a network adhering to a scale-free distribution, we screened for the optimal soft-thresholding power. As illustrated in Figures 3A,D, when the soft thresholds for the AKI and sepsis datasets were set to 5 and 7, respectively, the scale-free topology fit index reached 0.9, ensuring the biological relevance of the networks. Based on these parameters, distinct co-expression modules were identified using the dynamic tree cut algorithm, as shown in the hierarchical clustering dendrograms (Figures 3B,E).

Subsequently, to determine the key modules most relevant to T-cell immune status, we calculated the correlation between module eigengenes and the infiltration abundance/function of various T-cell subtypes. In the AKI dataset (Figure 3C), the yellow, pink, blue, black, and red modules exhibited the strongest positive correlations with multiple T-cell subtypes. Similarly, in the sepsis dataset (Figure 3F), lightcyan, tan, green, and midnightblue were identified as key modules highly positively correlated with T-cell infiltration. To unearth the shared molecular signatures linking the immune mechanisms of AKI and sepsis, we intersected the genes within the significantly positively correlated modules from both conditions. As shown in the Venn diagram in (Figure 3G, 24 shared genes were ultimately identified (Supplementary Table S2). These genes are considered potential core regulators driving T-cell dysfunction in both diseases and were utilized for subsequent analyses.

### 3.3 Screening of hub genes and construction of diagnostic models

**FIGURE 4:**
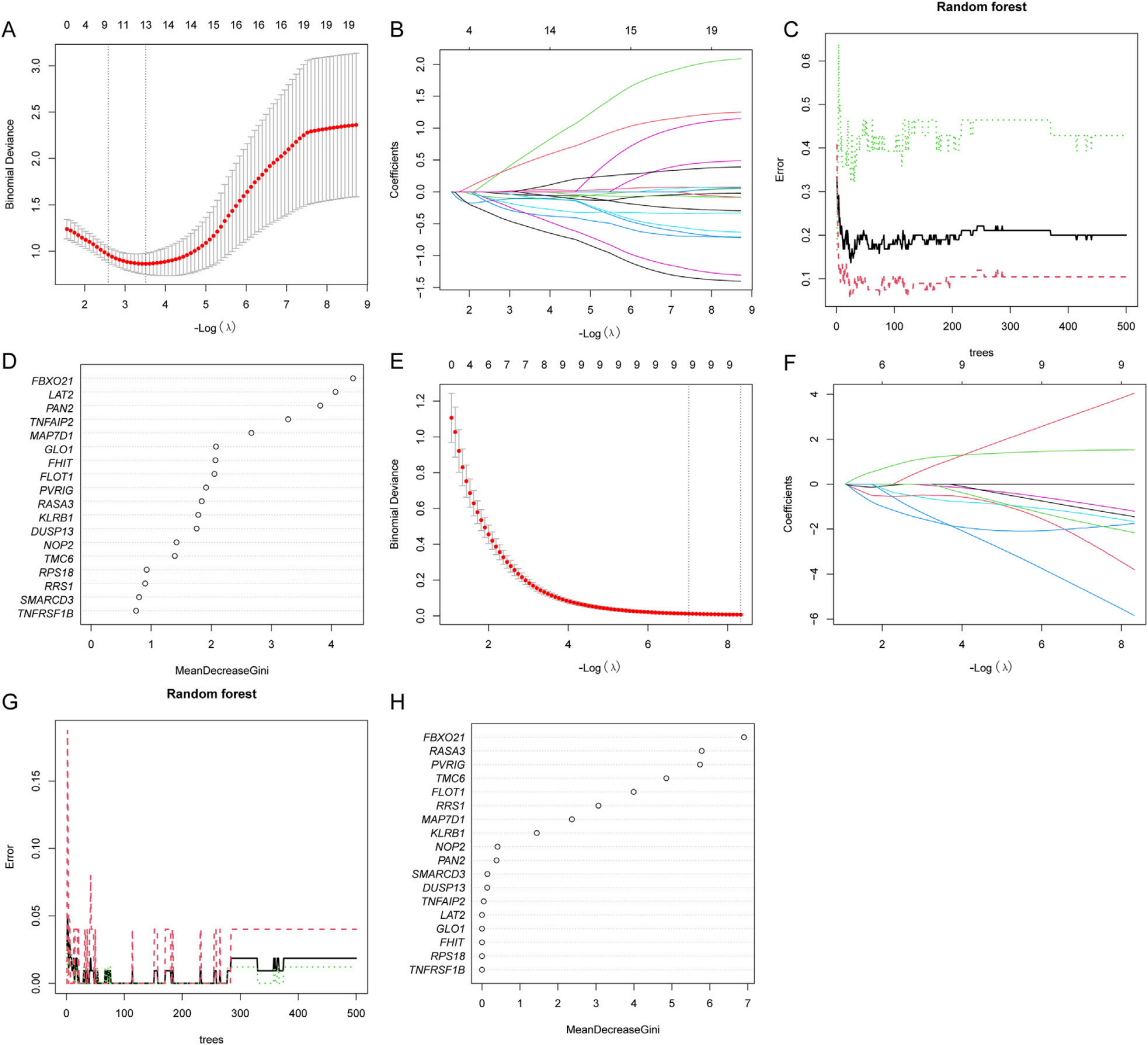
Selection of key diagnostic feature genes for AKI and sepsis via LASSO regression and Random Forest algorithms. **(A,B)** Feature selection via the LASSO algorithm in the AKI dataset. **(A)** Selection of the optimal parameter λ using 10-fold cross-validation. The vertical dashed line indicates the λ value where the binomial deviance is minimized. **(B)** LASSO coefficient profiles illustrating the shrinkage of regression coefficients for each gene with varying λ values. **(C,D)** Feature selection via the Random Forest algorithm in the AKI dataset. **(C)** The relationship between prediction error and the number of decision trees in the Random Forest model. **(D)** Variable importance plot ranking the top key genes based on the Mean Decrease Gini. **(E,F)** Feature selection via the LASSO algorithm in the sepsis dataset. **(E)** Cross-validation error plot for parameter selection. **(F)** LASSO coefficient profiles of the genes. **(G,H)** Feature selection via the Random Forest algorithm in the sepsis dataset. **(G)** Relationship between error rates and the number of trees. **(H)** Ranking of gene importance based on the Mean Decrease Gini.

**FIGURE 5:**
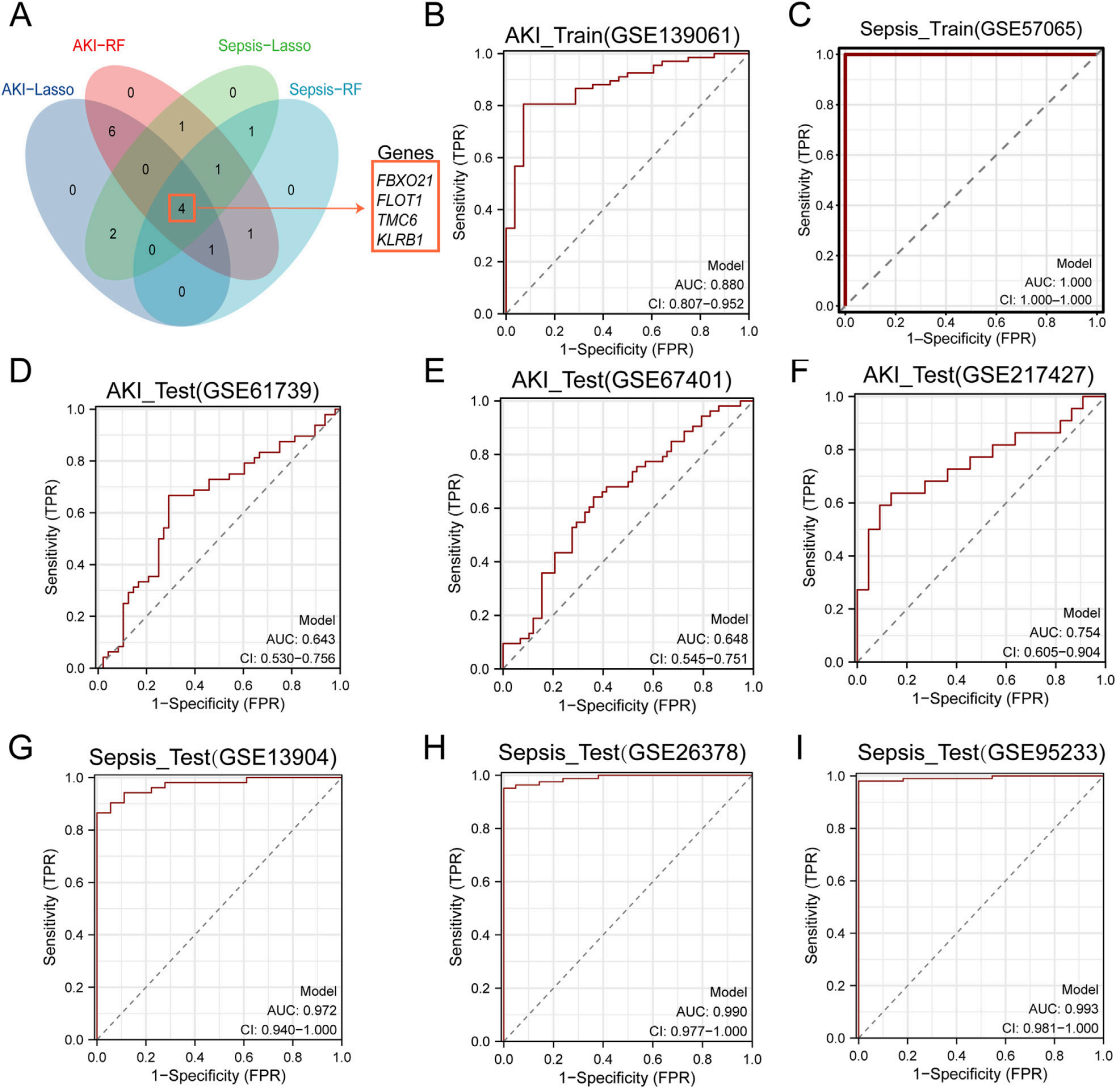
Identification of shared diagnostic genes and construction and validation of diagnostic models across multiple datasets. **(A)** Venn diagram displaying the intersection of feature genes selected by LASSO and RF algorithms within the AKI and sepsis datasets. **(B–I)** ROC curve analysis of the combined diagnostic gene model for predicting acute kidney injury and sepsis. **(B)** The AKI training set (GSE139061). **(C)** The sepsis training set (GSE57065). **(D–F)** Three independent AKI external validation cohorts: GSE61739 **(D)**, GSE67401 **(E)**, and GSE217427 **(F)**. **(G–I)** Three independent sepsis external validation cohorts: GSE13904 **(G)**, GSE26378 **(H)**, and GSE95233 **(I)**. The Area Under the Curve (AUC) and 95% Confidence Interval (CI) are annotated in the plots.

To screen for biomarkers with robust diagnostic value for both AKI and sepsis, we integrated LASSO regression and Random Forest algorithms for feature selection. LASSO regression was first performed independently in the AKI and sepsis datasets, and the optimal penalty parameter λ was determined by 10-fold cross-validation (Figures 4A,B,E,F). Random Forest analysis was then used to rank candidate genes by importance (Figures 4C,D,G,H). By intersecting the genes selected by both algorithms in both disease settings, four shared hub genes were identified: *FBXO21*, *FLOT1*, *TMC6*, and *KLRB1* (Figure 5A). These four genes may represent complementary biological dimensions of the shared host response in AKI and sepsis (Supplementary Table S4). *FLOT1* is related to lipid raft organization and inflammatory signal transduction, suggesting enhanced membrane-associated immune signaling. *FBXO21* is involved in ubiquitin-mediated protein turnover, implying altered proteostasis during critical illness. *KLRB1* is associated with cytotoxic lymphocyte and NK/T-cell states, linking the signature to adaptive immune dysregulation. *TMC6* may reflect membrane-associated immune regulation and cellular stress responses. Therefore, the diagnostic signature is not merely a statistical classifier but may capture convergent inflammatory, immune, and stress-response programs shared by AKI and sepsis.

Based on these four hub genes, a multivariate logistic regression diagnostic model was constructed and evaluated using ROC analysis (Supplementary Table S5). In the AKI dataset, the model demonstrated good diagnostic efficacy in the training set (GSE139061) (AUC = 0.880, Figure 5B). In the three external validation cohorts, the model maintained consistent predictive capability (AUC range: 0.643–0.754, Figures 5D–F), demonstrating its robustness across different clinical contexts. For sepsis, the four-gene logistic regression model achieved an apparent AUC of 1.000 in the training cohort GSE57065 (Figure 5C). Because this value was obtained from the model-development cohort, it should be interpreted cautiously and may partly reflect optimistic performance estimation. To further assess the generalizability of the model, we evaluated it in three independent external sepsis cohorts. The model maintained strong diagnostic performance, with AUC values of 0.972 in GSE13904 (Figure 5G), 0.990 in GSE26378 (Figure 5H), and 0.993 in GSE95233 (Figure 5I). These independent validation results suggest that the four-gene signature captures a reproducible sepsis-associated transcriptomic signal. Nevertheless, the perfect apparent AUC in the training cohort should not be overinterpreted as evidence of perfect diagnostic accuracy.

### 3.4 Construction and clinical evaluation of shared diagnostic nomograms

**FIGURE 6:**
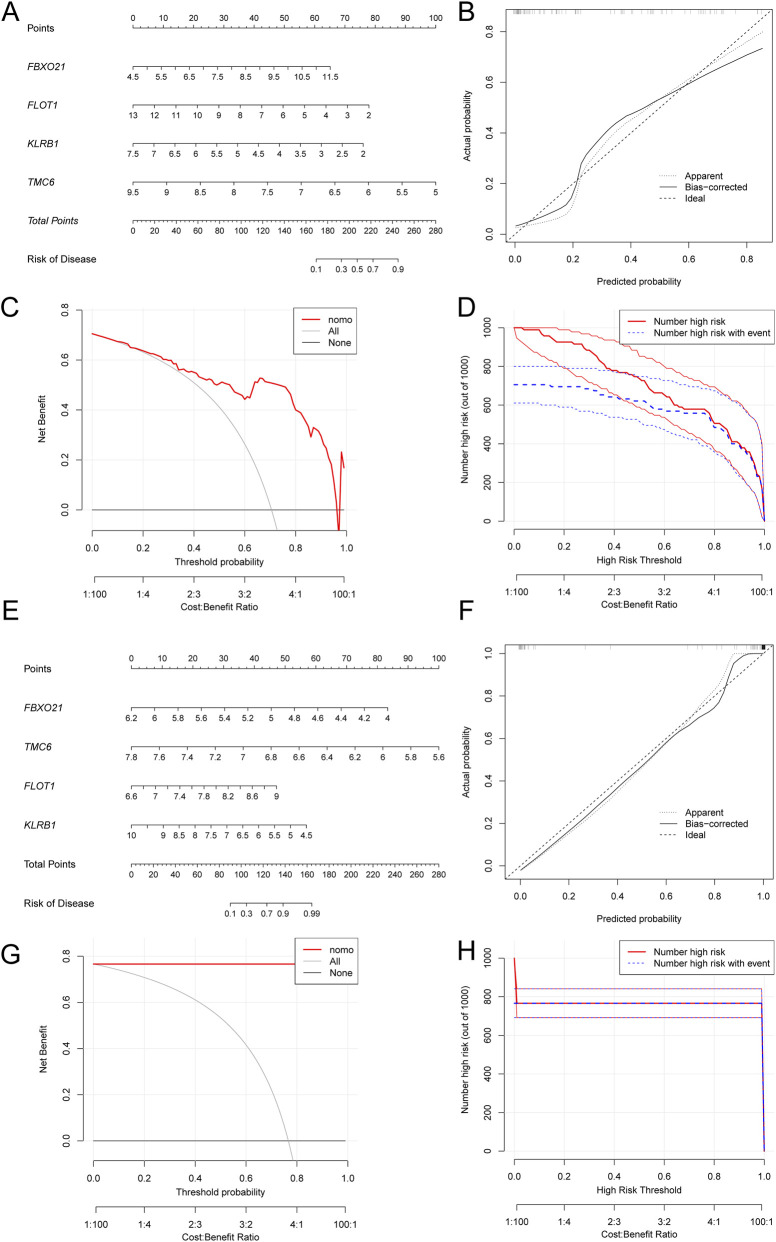
Construction and evaluation of shared hub gene-based diagnostic nomograms for AKI and sepsis. **(A–D)** Construction and performance evaluation of the diagnostic model for the AKI group. **(A)** Diagnostic nomogram constructed using the four identified hub genes (*FBXO21*, *FLOT1*, *KLRB1*, *TMC6*). Each gene corresponds to specific points on the scale according to its expression level; the total points (sum of individual scores) map to the predicted probability of disease for an individual. **(B)** Calibration curve assessing the predictive accuracy of the nomogram. The x-axis represents the predicted risk, and the y-axis denotes the actual observed risk. The diagonal dashed line signifies the ideal prediction model, while the solid line represents the bias-corrected performance via bootstrapping; closer alignment between the two indicates superior calibration. **(C)** Decision Curve Analysis (DCA) evaluating the clinical net benefit. The red curve represents the nomogram model, while the gray and black lines represent the “Treat-all” and “Treat-none” strategies, respectively. **(D)** Clinical Impact Curve (CIC) assessing the clinical utility of the model. The red curve indicates the number of individuals deemed high-risk by the model at various threshold probabilities, while the blue curve represents the number of true positive cases. **(E–H)** Construction and evaluation of the diagnostic model for the sepsis group, comprising the nomogram **(E)**, calibration curve **(F)**, DCA curve **(G)**, and CIC **(H)**.

To facilitate the quantitative assessment of disease risk by clinicians, we constructed diagnostic nomograms for AKI and sepsis, respectively, based on the four identified hub genes (Figures 6A,E). Within these models, the expression level of each gene is assigned a specific score, and the total score allows for an intuitive prediction of an individual’s probability of developing the disease. To validate the clinical utility of the nomograms, we conducted a comprehensive multi-dimensional evaluation. Calibration curves demonstrated good agreement between the predicted curves and the ideal curves in both the AKI (Figure 6B) and sepsis (Figure 6F) models, indicating high consistency between the predicted probabilities and actual observations. DCA revealed that, across a wide range of threshold probabilities, the net clinical benefit of using the nomogram models for intervention was significantly superior to the “treat-all” or “treat-none” strategies (Figures 6C,G). Furthermore, Clinical Impact Curves (CIC) further corroborated the high concordance between the population deemed high-risk by the model and the actual number of positive cases (Figures 6D,H).

### 3.5 Expression landscape of hub genes in single cells

**FIGURE 7:**
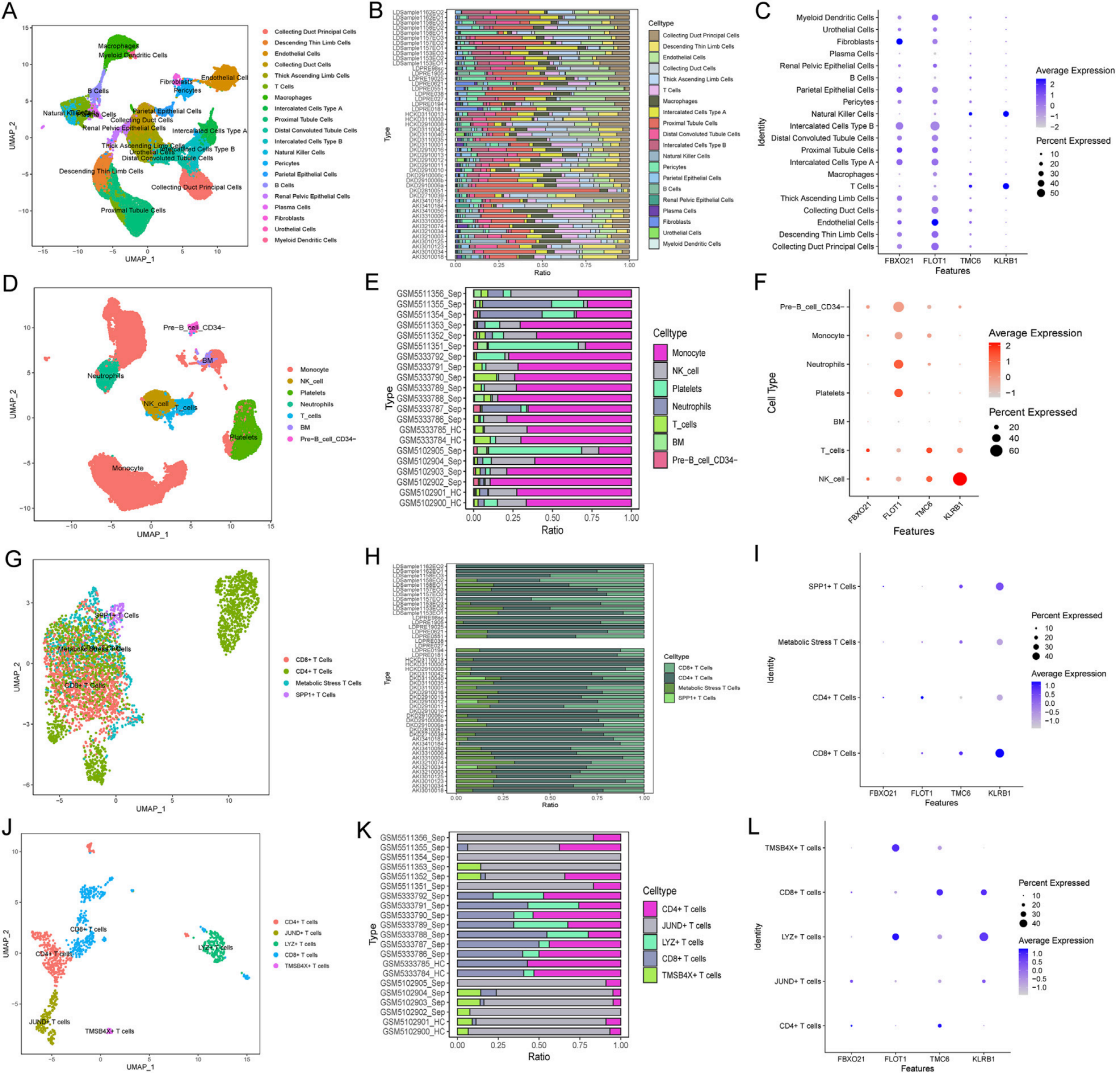
Single-cell RNA sequencing landscape and cell-specific expression analysis of shared hub genes in AKI and sepsis. **(A–C)** Characteristics of the single-cell atlas in the AKI dataset. **(A)** UMAP dimensionality reduction plot of all cell types, visualizing major cell populations including renal tubular epithelial cells and immune cells. **(B)** Stacked bar plot illustrating the relative abundance proportions of cell types across different samples. **(C)** Bubble plot displaying the expression profiles of the four shared hub genes (*FBXO21*, *FLOT1*, *TMC6*, *KLRB1*) in major cell types of AKI. The size of the dot corresponds to the percentage of cells expressing the gene, while the color intensity indicates the average expression level. **(D–F)** Characteristics of the single-cell atlas in the sepsis dataset. **(D)** UMAP plot of major cell types in peripheral blood, including neutrophils, monocytes, and T cells. **(E)** Proportional distribution of cell types in sepsis samples compared to control samples. **(F)** Bubble plot showing the expression of shared hub genes in major cell types of sepsis. **(G–I)** Re-clustering analysis of T-cell subsets in AKI. **(G)** UMAP plot of T-cell subsets, identifying four distinct clusters: *CD4* ^+^ T cells, *CD8* ^+^ T cells, Metabolic Stress T cells, and *SPP1* ^+^ T cells. **(H)** Proportions of each T-cell subset across samples. **(I)** Expression characteristics of hub genes within different T-cell subsets of AKI. **(J–L)** Re-clustering analysis of T-cell subsets in sepsis. **(J)** UMAP plot of T-cell subsets, identifying characteristic clusters including *CD4* ^+^, *CD8* ^+^, as well as *JUND* ^+^, *LYZ* ^+^, and *TMSB4X* ^+^ T-cell subsets. **(K)** Proportional distribution of each T-cell subset. **(L)** Expression characteristics of hub genes within different T-cell subsets of sepsis.

To further determine the cellular origin of the shared hub genes, we analyzed scRNA-seq datasets from AKI and sepsis. In the AKI dataset, the major cell populations included renal tubular epithelial cells, T cells, and NK cells (Figures 7A,B). In the sepsis dataset, the cellular composition was dominated by peripheral blood immune cells, including neutrophils, monocytes, and T cells (Figures 7D,E). Bubble plot analysis revealed that *KLRB1* and *TMC6* were preferentially expressed in T cells and NK cells in both AKI and sepsis, whereas *FLOT1* showed broader expression across multiple cell types (Figures 7C,F). These single-cell findings provide important biological context for the bulk transcriptomic signature. The broad distribution of *FLOT1* suggests that inflammatory signal amplification may occur across multiple cell compartments. In contrast, the preferential expression of *KLRB1* and *TMC6* in T/NK-lineage cells indicates that the shared diagnostic signature is closely linked to lymphocyte remodeling rather than representing only nonspecific tissue injury. Therefore, the four-gene model likely reflects both disease-associated cellular composition changes and intrinsic transcriptional alterations within immune cell populations. Given the strong association between the hub genes and T-cell-related modules in WGCNA, we further re-clustered T cells in AKI and sepsis. In AKI, T-cell subsets included *CD4* ^+^ T cells, *CD8* ^+^ T cells, Metabolic Stress T cells, and *SPP1* ^+^ T cells (Figures 7G,H). *KLRB1* was enriched in *CD8* ^+^ T cells and Metabolic Stress T cells (Figure 7I), suggesting that cytotoxic and stress-adapted T cells may participate in the renal inflammatory microenvironment. In sepsis, T-cell subsets included *CD4* ^+^, *CD8* ^+^, *JUND* ^+^, *LYZ* ^+^, and *TMSB4X* ^+^ T-cell populations (Figures 7J,K), with *KLRB1* and *TMC6* predominantly enriched in CD8^+^ T cells (Figure 7L). Collectively, these results suggest that CD8^+^ T-cell remodeling is a common feature of both diseases, but the local renal environment in AKI may impose additional metabolic stress on infiltrating T cells.

### 3.6 High correlation with *CD8* ^+^ T Cells and differential functional enrichment of hub genes

**FIGURE 8:**
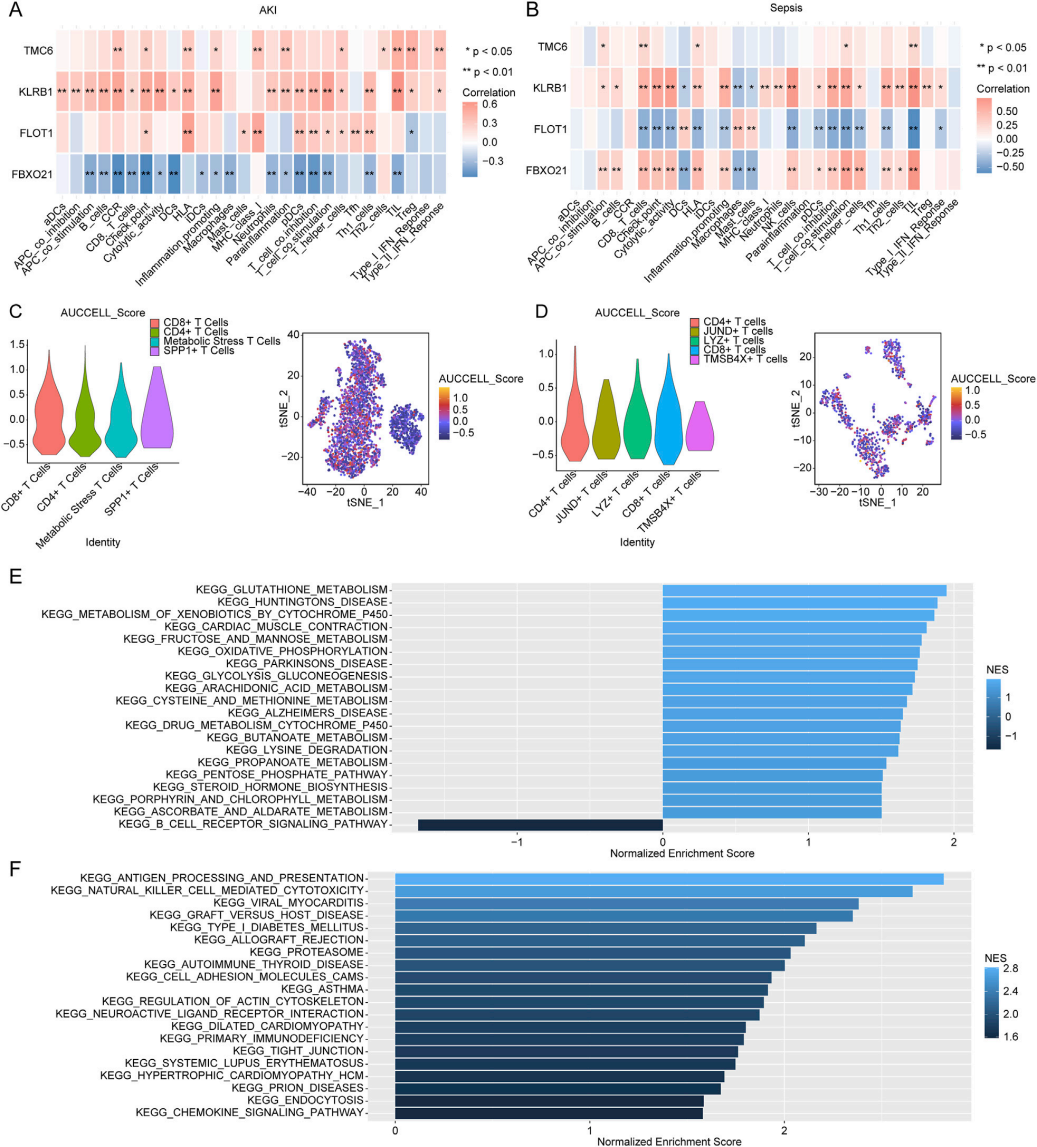
Correlation verification between Hub genes and immune infiltration and functional enrichment analysis. **(A,B)** Heatmaps illustrating the correlation between the infiltration abundance of immune cell subtypes (evaluated via the ssGSEA algorithm) and Hub gene expression levels. **(A)** The AKI group; **(B)** The sepsis group. The color scale represents the Spearman correlation coefficient (red indicates positive correlation; blue indicates negative correlation). Asterisks denote statistical significance (P < 0.05, *P < 0.01). **(C,D)** Evaluation of Hub gene set activity scores across different T-cell subtypes at the single-cell level using *AddModuleScore* function. The left panels are violin plots displaying the distribution of scores across subpopulations; the right panels are feature plots mapping these scores onto the 2D dimensionality reduction space. **(C)** In the AKI group, *CD8* ^+^ T cells exhibited the highest Hub gene activity scores. **(D)** In the sepsis group, *CD8* ^+^ T cells similarly displayed specifically high activity scores. **(E)** GSEA enrichment analysis performed on *CD4* ^+^ T cells in the AKI group, highlighting significantly enriched KEGG pathways primarily involving metabolic processes such as Oxidative phosphorylation, Glutathione metabolism, and Glycolysis/Gluconeogenesis. **(F)** GSEA enrichment analysis performed on *CD8* ^+^ T cells in the sepsis group, where significantly enriched pathways mainly include immune response pathways such as Antigen processing and presentation, Natural killer cell-mediated cytotoxicity, and Graft-versus-host disease.

Immune infiltration correlation analysis based on ssGSEA showed that hub gene expression was positively associated with T-cell abundance and cytotoxic immune activity in both AKI and sepsis (Figures 8A,B). These associations indicate that the diagnostic signature is closely linked to adaptive immune remodeling, especially cytotoxic lymphocyte-related programs. At single-cell resolution, *AddModuleScore* analysis further confirmed that the hub-gene signature was highly active in T-cell subsets. In AKI, *CD8* ^+^ T cells exhibited the highest hub-gene activity scores (Figure 8C). In sepsis, *CD8* ^+^ T cells also showed particularly high hub-gene signature activity (Figure 8D). This consistency between bulk immune correlation and single-cell scoring supports the biological relevance of the four-gene signature and suggests that *CD8* ^+^ T cells may be a central cellular compartment connecting sepsis and AKI. GSEA revealed disease-specific functional patterns within T-cell subsets. In AKI, T-cell-related genes were enriched in metabolic pathways, including oxidative phosphorylation, glutathione metabolism, and glycolysis/gluconeogenesis (Figure 8E). These pathways suggest that T cells in the injured kidney may undergo metabolic reprogramming to adapt to hypoxia, oxidative stress, and nutrient limitation within the damaged renal microenvironment. However, sustained metabolic stress may impair effector function and contribute to maladaptive inflammation or tissue injury. In contrast, *CD8* ^+^ T cells in sepsis were enriched for immune effector pathways, including antigen processing and presentation, natural killer cell-mediated cytotoxicity, and graft-versus-host disease-related pathways (Figure 8F). These pathways indicate enhanced cytotoxic and antigen-response programs, consistent with systemic immune activation during infection. Together, these findings suggest that although AKI and sepsis share a T-cell-associated hub-gene signature, the functional state of T cells diverges between the two conditions: AKI is characterized by metabolic stress adaptation, whereas sepsis is characterized by cytotoxic activation accompanied by potential immune exhaustion.

### 3.7 Deciphering intercellular communication networks among T-cell subtypes in AKI and sepsis microenvironments

**FIGURE 9:**
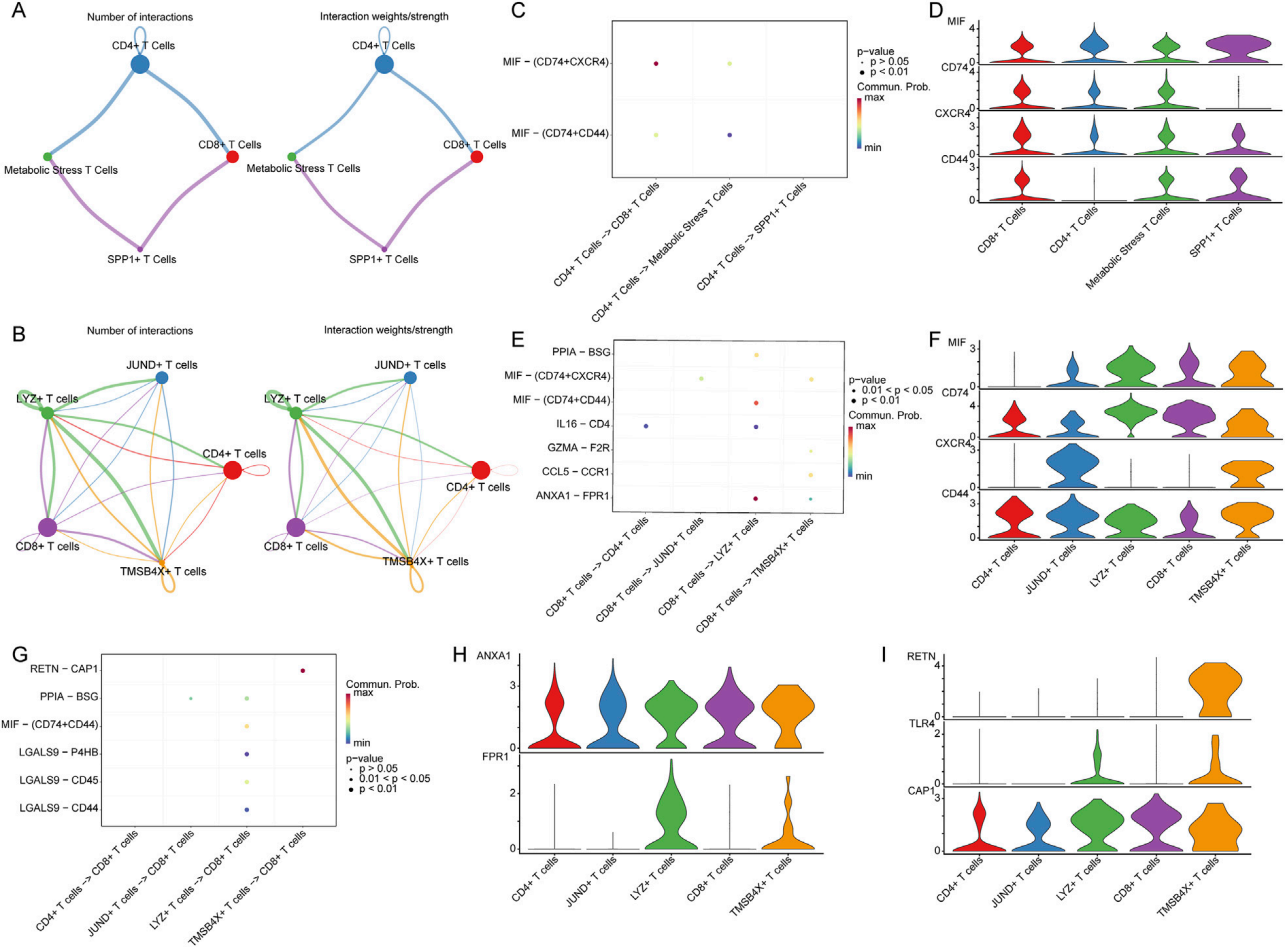
Analysis of intercellular communication networks among T-cell subtypes within the AKI and sepsis microenvironments. **(A,B)** Circle plots (Chord diagrams) of the cell-cell communication networks, illustrating the number of inferred interactions (left panel) and interaction strength (right panel) among T-cell subtypes in AKI **(A)** and sepsis **(B)**. The thickness of the connecting lines represents the strength of the interaction signal. **(C)** Bubble plot displaying significant ligand-receptor pairs in AKI, with *CD4* ^+^ T cells acting as the ligand source (sender) targeting other T-cell subtypes. The color gradient represents communication probability, and the dot size indicates the statistical significance (P-value). **(D,F)** Violin plots showing the expression distribution of genes related to the *MIF* signaling pathway (ligand *MIF* and its receptors CD74, CXCR4, CD44) across T-cell subtypes in AKI **(D)** and sepsis **(F)**. **(E)** Bubble plot illustrating significant signaling interactions in sepsis with *CD8* ^+^ T cells as the sender, primarily involving pathways such as *ANXA1* and *MIF*. **(G)** Bubble plot displaying signaling interactions in sepsis with *CD8* ^+^ T cells as the receiver, highlighting incoming signals such as *RETN* and *LGALS9*. **(H)** Violin plots showing the expression profile of the *ANXA1* signaling pathway across T-cell subtypes in sepsis. **(I)** Violin plots demonstrating the expression characteristics of the RESISTIN (*RETN*) signaling pathway in sepsis.

To further explore the molecular interactions underlying these functional differences, we performed CellChat analysis to infer intercellular communication networks among T-cell subsets. In AKI, *CD4* ^+^ T cells were predicted to act as a major source of outgoing signals (Figures 9A,C). Among the inferred ligand-receptor interactions, the *MIF* signaling pathway was prominent. *CD4* ^+^ T cells expressed *MIF*, which was predicted to interact with CD74/CXCR4 and CD74/CD44 receptor complexes on *CD8* ^+^ T cells and Metabolic Stress T cells (Figure 9D). This inferred communication pattern provides a potential mechanistic explanation for the metabolic phenotype observed in AKI-associated T cells. *MIF* is known to participate in inflammatory activation and metabolic regulation. Therefore, *CD4* ^+^ T-cell-derived *MIF* signaling may contribute to metabolic remodeling of *CD8* ^+^ T cells in the injured kidney. Such remodeling may initially support cell survival in a hypoxic and inflammatory environment but could become maladaptive if prolonged, thereby aggravating local immune-mediated tissue injury.

In sepsis, the communication network was more complex and appeared to center around *CD8* ^+^ T cells (Figure 9B). As predicted signal senders, *CD8* ^+^ T cells were associated with *ANXA1*-related signaling (Figure 9E). The inferred *ANXA1*–*FPR1* interaction may reflect an endogenous feedback mechanism involved in regulating leukocyte migration, inflammatory resolution, or cytotoxic immune activity (Figure 9H). As predicted signal receivers, *CD8* ^+^ T cells received *RETN*-related signaling, primarily from *LYZ* ^+^ T cells through the *RETN*–*CAP1* ligand-receptor pair (Figures 9G,I). Because *RETN* was also among the strongly upregulated genes in the sepsis differential gene expression analysis, this inferred resistin-associated signal may represent an important link between innate inflammatory activation and *CD8* ^+^ T-cell dysfunction. These findings suggest that sepsis-associated *CD8* ^+^ T cells may be shaped by inflammatory ligand inputs from myeloid-like immune populations, potentially contributing to cytotoxic activation followed by immune exhaustion. Thus, AKI and sepsis may share a *CD8* ^+^ T-cell-centered immune signature but differ in upstream regulatory signals: *MIF*-associated metabolic stress in AKI and *RETN*-associated inflammatory modulation in sepsis.

### 3.8 Experimental validation of hub gene expression trends

**FIGURE 10:**
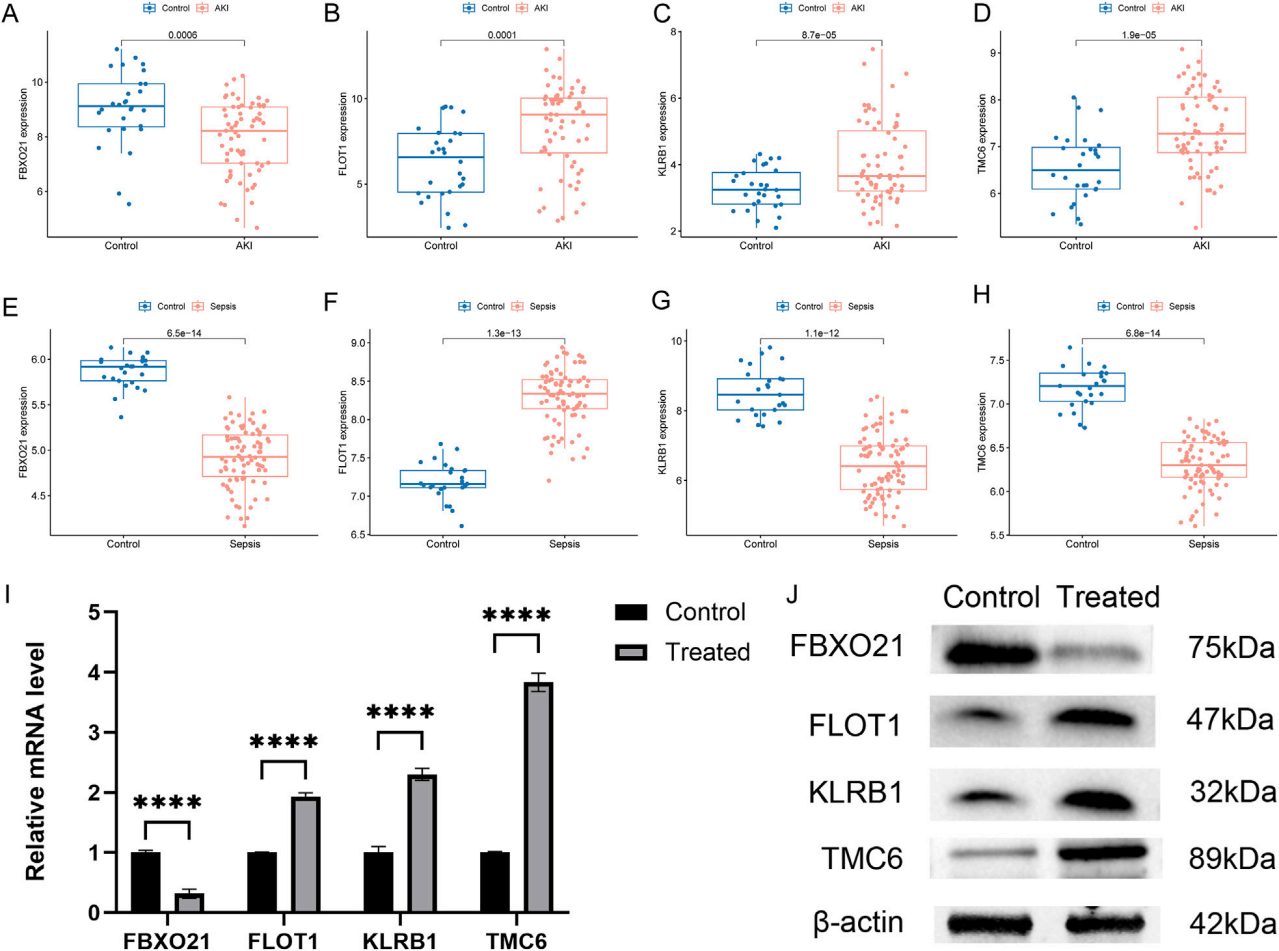
Validation of expression levels of the four shared hub genes in AKI and sepsis datasets. **(A–D)** Boxplots illustrating the differential gene expression of the four hub genes between AKI samples and controls in the AKI dataset: **(A)** *FBXO21*, **(B)** *FLOT1*, **(C)** *KLRB1*, and **(D)** *TMC6*. **(E–H)** Boxplots illustrating the differential expression of the four hub genes between sepsis samples and controls in the sepsis dataset: **(E)** *FBXO21*, **(F)** *FLOT1*, **(G)** *KLRB1*, and **(H)** *TMC6*. The horizontal line within each box represents the median, and the upper and lower boundaries of the box denote the interquartile range (IQR). The values displayed above the plots indicate the P-values derived from statistical significance testing (all P < 0.001). **(I,J)** The qRT-PCR and WB trends of hub genes in the control group and the sepsis group.

The four genes exhibited highly consistent patterns of dysregulation across both disease cohorts (Figure 10), with differences reaching high statistical significance (P < 0.001). Specifically, compared to normal controls, *FLOT1* displayed a significant trend of upregulation in both AKI (Figure 10B) and sepsis (Figure 10F) samples. Conversely, *FBXO21* demonstrated significant downregulation in both pathological states (Figures 10A,E). The remaining two genes exhibited opposing expression trends between the two diseases (Figures 10C,D and 10G-H). Further verification of the expression trends of *FBXO21*, *FLOT1*, *KLRB1* and *TMC6* in the control group and the sepsis group was conducted through qRT-PCR and WB experiments (Figures 10I,J).

## 4 Discussion

AKI and sepsis are inextricably intertwined clinical syndromes, frequently forming a vicious cycle characterized by local tissue injury and systemic immune dysregulation (Pais et al., 2024). Despite their etiological correlation, the molecular disparities between kidney-specific responses and systemic immune responses remain poorly understood. In this study, we integrated transcriptomic approaches-ranging from bulk transcriptomics to single-cell resolution-to systematically dissect the commonalities and specificities of AKI and sepsis. We identified a robust four-gene diagnostic signature (*FLOT1*, *FBXO21*, *KLRB1*, *TMC6*). More importantly, our findings suggest that AKI may be characterized by *MIF*-axis-associated T-cell metabolic reprogramming, whereas sepsis may be dominated by a Resistin-signal-associated cytotoxic and exhausted T-cell phenotype.

This study precisely identified four core genes (*FLOT1*, *FBXO21*, *KLRB1*, and *TMC6*) from bulk expression profiles using machine-learning algorithms, and this molecular signature demonstrated excellent diagnostic performance across multiple external validation cohorts. More importantly, through deep integration with scRNA-seq data, the study further localized this signature to specific *CD8* ^+^ T-cell subsets. This strategy, moving from global transcriptomic screening to precise single-cell localization, not only confirms that the biomarker captures essential changes in the immune microenvironment, but also reveals the distinct functional logic of these genes in AKI and sepsis, thereby organically integrating mathematical prediction with biological mechanisms. Among them, *FLOT1* exhibited consistent upregulation in both AKI and sepsis. *FLOT1* is a pivotal component of lipid rafts, essential for signal transduction and receptor stabilization. A previous study demonstrated that *FLOT1* facilitates TNF-α receptor signaling and sustains NF-κB activation, a mechanism that may exacerbate inflammatory responses during tissue injury (Li et al., 2025; Nian et al., 2025; Song et al., 2012). Genes such as *SLC2A3* (Hochrein et al., 2022), and *IL7R* (Winer et al., 2022) are associated with glucose metabolism, T-cell receptor signaling, or neutrophil activation pathways, all of which are dysregulated in both acute kidney injury and sepsis. These findings further support the robustness and biological relevance of our multi-algorithm feature selection strategy. Consistent with recent integrative and machine-learning studies of sepsis-associated AKI, these markers add to a rapidly expanding body of work on transcriptomic biomarker discovery in this setting (Chen et al., 2025; Chen et al., 2024).

In contrast, the expression trend in sepsis points towards the systemic mobilization and activation of cytotoxic T cells in peripheral blood (Jensen et al., 2018). This finding aligns with our scRNA-seq analysis, where *KLRB1* specifically marked *CD8* ^+^ T cells, suggesting that this expression disparity stems from the distinct fates of T cells in local tissues versus the systemic circulation. In AKI, we identified a unique Metabolic Stress T-cell population (Zhou et al., 2022). GSEA and CellChat analyses suggested that these cells may be influenced by an inferred *MIF*-CD74/CXCR4 axis originating from *CD4* ^+^ T cells (Pais et al., 2024) that not only promotes inflammation but also regulates glucose uptake and glycolysis by stabilizing HIF-1α (Li et al., 2018). Conversely, in sepsis, the T-cell landscape may be shaped by inferred Resistin (*RETN*) signaling derived from *LYZ* ^+^ myeloid-like T cells (Sundén-Cullberg et al., 2007). High serum resistin levels correlate with sepsis severity (Hotchkiss et al., 2013).

Finally, we translated these molecular findings into potential clinical tools. The nomograms constructed based on the four hub genes demonstrated excellent predictive performance, particularly in sepsis cohorts, in which the four-gene model achieved extremely high AUC values. From a clinical perspective, this model may provide a unified host immune response-based assessment tool for sepsis and AKI, two conditions that are often difficult to distinguish in ICU settings. Compared with conventional functional indicators such as serum creatinine, which are influenced by multiple confounding factors and exhibit a well-recognized lag effect, this transcriptome-based nomogram may enable earlier identification of immune dysregulation. Such an early-warning framework could help clinicians move from disease-specific treatment toward mechanism-guided precision intervention, for example, by determining whether immunomodulatory or metabolic support therapies should be initiated based on molecular features. Despite these promising findings, several limitations should be acknowledged. First, although the four-gene model achieved an apparent AUC of 1.000 in the sepsis training cohort, this result should be interpreted with caution, as it may reflect optimistic performance estimation or potential overfitting in the model development dataset. Nevertheless, the model maintained high AUC values in three independent external sepsis validation cohorts, supporting the robustness and potential diagnostic value of this gene signature. Further prospective, multicenter studies with larger sample sizes are still required to validate its diagnostic performance and clinical applicability. Second, although microarray technology has been widely used in meta-analyses, an inherent limitation is that it can only capture probe information predefined by specific platforms and may therefore miss certain undefined non-coding RNAs, resulting in reduced transcriptomic coverage compared with whole-transcriptome sequencing. In addition, the metabolic remodeling of CD8^+^ T cells in AKI identified in this study further extends recent insights into the immune microenvironment of S-AKI, such as those described in Ye et al. (2025). These findings suggest that future studies should pay attention not only to the burst of inflammatory mediators but also to the survival strategies adopted by immune cells under local tissue stress. Therefore, future investigations integrating whole-transcriptome sequencing, preclinical models, and prospective clinical cohorts are warranted to further validate potential regulatory factors not covered by microarray platforms and to confirm the translational value of the proposed molecular signatures.

## 5 Conclusion

By integrating machine-learning algorithms with transcriptomic analyses, this study systematically identified and computationally validated a shared four-gene diagnostic signature comprising FBXO21, FLOT1, TMC6, and KLRB1. The signature demonstrated excellent and highly consistent discriminatory performance across the sepsis validation cohorts (AUC 0.972–0.993), whereas its performance in the external AKI cohorts was more modest and variable (AUC 0.643–0.754). This differential indicates that the signature functions as a robust diagnostic classifier for sepsis but provides only moderate discriminatory capacity for AKI in its current form, a distinction that must be borne in mind when considering clinical application. Single-cell resolution further revealed that this molecular signature primarily reflected the heterogeneous evolution of CD8^+^ T cells during systemic infection and local organ injury, namely, a MIF-axis-driven metabolic stress phenotype in AKI and a Resistin-signaling-dominated cytotoxic/exhausted phenotype in sepsis. Taken together, these findings support the four-gene signature as a promising early molecular diagnostic tool that is reliable for sepsis, while for sepsis-associated kidney injury it still requires further refinement and optimization before robust diagnostic performance can be claimed. Beyond its diagnostic implications, the study clarifies the potential role of immune-metabolic interactions in disease progression, thereby offering candidate biological targets for the future development of targeted immunomodulatory therapies.

These conclusions should nonetheless be interpreted with considerable caution and within several important constraints of the present study. First, the results are derived primarily from retrospective, publicly available transcriptomic datasets; the bulk expression data were largely obtained from microarray platforms with limited transcriptomic coverage; and the single-cell cohorts were relatively small, which may in part account for the weaker and less stable AKI validation performance. Second, and importantly, the present *in vitro* experiments do not yet constitute conclusive experimental validation of the expression trends: because of the inconsistency between the Sections 2, 3 described above, the current qPCR and Western blot data cannot be regarded as fully reliable, and the expression changes of the four hub genes therefore remain to be confirmed by rigorously designed and internally consistent experiments. Accordingly, the diagnostic value of the four-gene signature, particularly in the AKI setting, together with the proposed immune-metabolic mechanisms, warrants confirmation in prospective, multicenter clinical cohorts and *in vivo* models, while the actual expression trends and the causal roles of the four hub genes remain to be firmly established through dedicated, methodologically robust functional experiments.

## Data Availability

The original contributions presented in the study are included in the article/Supplementary Material, further inquiries can be directed to the corresponding author.
