# Supplementary material for: Identification of common diagnostic biomarkers and immune landscapes in sepsis and acute kidney injury: a transcriptomic study integrating machine learning and single-cell analysis
Source: Front Genet. 2026 Jul 22;17:1837345. doi: 10.3389/fgene.2026.1837345 (PMC13436955; doi:10.3389/fgene.2026.1837345)
Supplement: Supplementary file 1 [file Image1.pdf]

Supplementary Materials

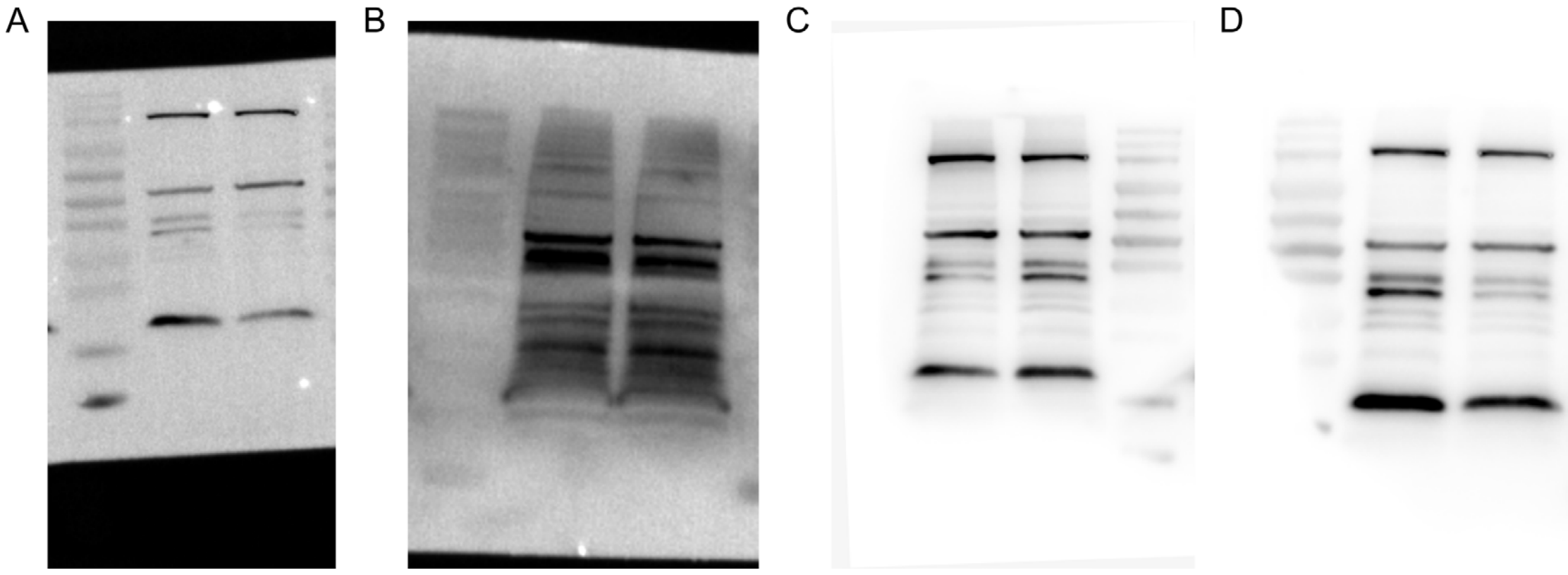

Figure S1 Original, uncropped Western blot images of the four shared hub gene proteins. Full, unadjusted membrane scans corresponding to the representative blots shown in Figure 10. (A) FLOT1. (B) FBXO21. (C) KLRB1. (D) TMC6. All images are presented in full, without cropping or contrast manipulation, and serve as the source data for the cropped panels shown in Figure 10.
